# Development of prognostic signature based on RNA binding proteins related genes analysis in clear cell renal cell carcinoma

**DOI:** 10.18632/aging.202360

**Published:** 2021-01-10

**Authors:** Qiang Chen, Zhi-Long Li, Sheng-Qiang Fu, Si-Yuan Wang, Yu-Tang Liu, Ming Ma, Xiao-Rong Yang, Wen-Jie Xie, Bin-Bin Gong, Ting Sun

**Affiliations:** 1 Department of Urology, The First Affiliated Hospital of Nanchang University, Nanchang 330006, Jiangxi Province, China

**Keywords:** clear cell renal cell carcinoma, RNA binding proteins, biomarker, prognostic model

## Abstract

RNA binding proteins (RBPs) play significant roles in the development of tumors. However, a comprehensive analysis of the biological functions of RBPs in clear cell renal cell carcinoma (ccRCC) has not been performed. Our study aimed to construct an RBP-related risk model for prognosis prediction in ccRCC patients. First, RNA sequencing data of ccRCC were downloaded from The Cancer Genome Atlas (TCGA) database. Three RBP genes (EIF4A1, CARS, and RPL22L1) were validated as prognosis-related hub genes by univariate and multivariate Cox regression analyses and were integrated into a prognostic model by least absolute shrinkage and selection operator (LASSO) Cox regression analysis. According to this model, patients with high risk scores displayed significantly worse overall survival (OS) than those with low risk scores. Moreover, the multivariate Cox analysis results indicated that risk score, tumor grade, and tumor stage were significantly correlated with patient OS. A nomogram was constructed based on the three RBP genes and showed a good ability to predict outcomes in ccRCC patients. In conclusion, this study identified a three-RBP gene risk model for predicting the prognosis of patients, which is conducive to the identification of novel diagnostic and prognostic molecular markers.

## INTRODUCTION

Renal cell carcinoma (RCC), one of the most common malignant urological tumors, accounts for approximately 2-3% of adult malignancies and can be divided into several pathological subtypes, such as clear cell renal cell carcinoma (ccRCC), chromophobe RCC and papillary RCC, among which the clear cell type accounts for approximately 80% of all RCCs [1, 2]. At present, partial nephrectomy is the best method for the treatment of localized ccRCC, but the incidence and mortality rates of metastatic and advanced ccRCC are still high because of their insensitivity to traditional chemoradiotherapy. Although new targeted therapies represent the current general trend for the treatment of advanced ccRCC, variable drug response rates and obvious side effects reflect the wide variability in the efficacy and survival benefits of these drugs [1]. Thus, investigating the underlying mechanisms of ccRCC progression and finding new biomarkers for early diagnosis and effective therapeutic targets are urgently needed.

RNA binding proteins (RBPs) are proteins that modulate gene expression by forming extensive protein-RNA interactions at the posttranscriptional level [3, 4]. They bind RNA transcripts in the 3′-untranslated region (3′-UTR) and exert essential roles in numerous biological processes, including RNA degradation, localization, turnover, storage, splicing, transport to the cytoplasm, and protein translation [5–7]. In total, 1542 RBPs were identified by genome-wide sequencing and bioinformatics analysis, which represent approximately seven percent of all protein-coding genes [3]. These RBPs were demonstrated to modulate molecular functions and biological processes such as RNA turnover, splicing, localization, and protein translation [8]. Furthermore, dysregulation of RBPs has been found in various human cancers, suggesting that RBPs may be reliable early molecular markers and therapeutic targets [9]. Thus, it is essential to investigate the regulatory mechanisms of RBPs in the initiation and progression of various cancers.

To date, numerous reports have indicated that RBPs are dysregulated in ccRCC tumorigenesis and are involved in RNA modification and turnover [10, 11]. For example, the RBP NONO binds to SKP2 and E2F8 mRNA to promote the proliferation of breast cancer at the posttranscriptional level [12]. Quaking (QKI) suppresses tumor proliferation and metastasis by downregulating the activity of Yes-associated protein in renal cancer [13]. SART3 binds to miR-34a to affect the cell cycle progression of lung cancer cells by downregulating miR-34a target genes CDK4/6 [14]. Taken together, the results of these studies demonstrate that individual RBPs can play essential biological roles in tumor progression. Furthermore, a comprehensive analysis of RBPs may assist us in sufficiently understanding the potential mechanisms that occur during cancer progression. Therefore, the ccRCC RNA sequencing dataset and corresponding clinical features were screened from The Cancer Genome Atlas (TCGA). In addition, a number of differentially expressed RBPs in normal and cancerous samples were identified via bioinformatics methods. Then, an effective gene signature was constructed using these RBPs to predict patient prognosis. In this study, we determined that multiple RBPs are related to the progression of ccRCC, which may be conducive to the identification of novel diagnostic and prognostic molecular markers.

## RESULTS

### Identification of differentially expressed RBPs in ccRCC

**Figure 1:**
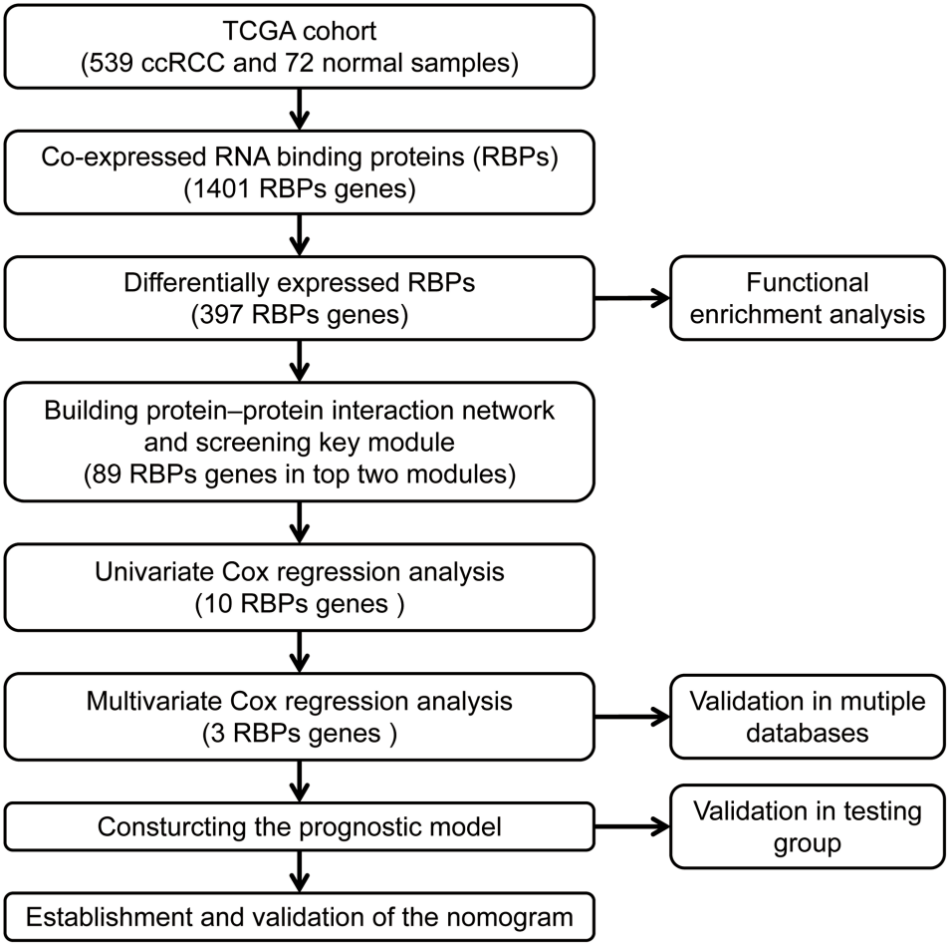
**The workflow for analyzing the RBPs in ccRCC.**

**Figure 2:**
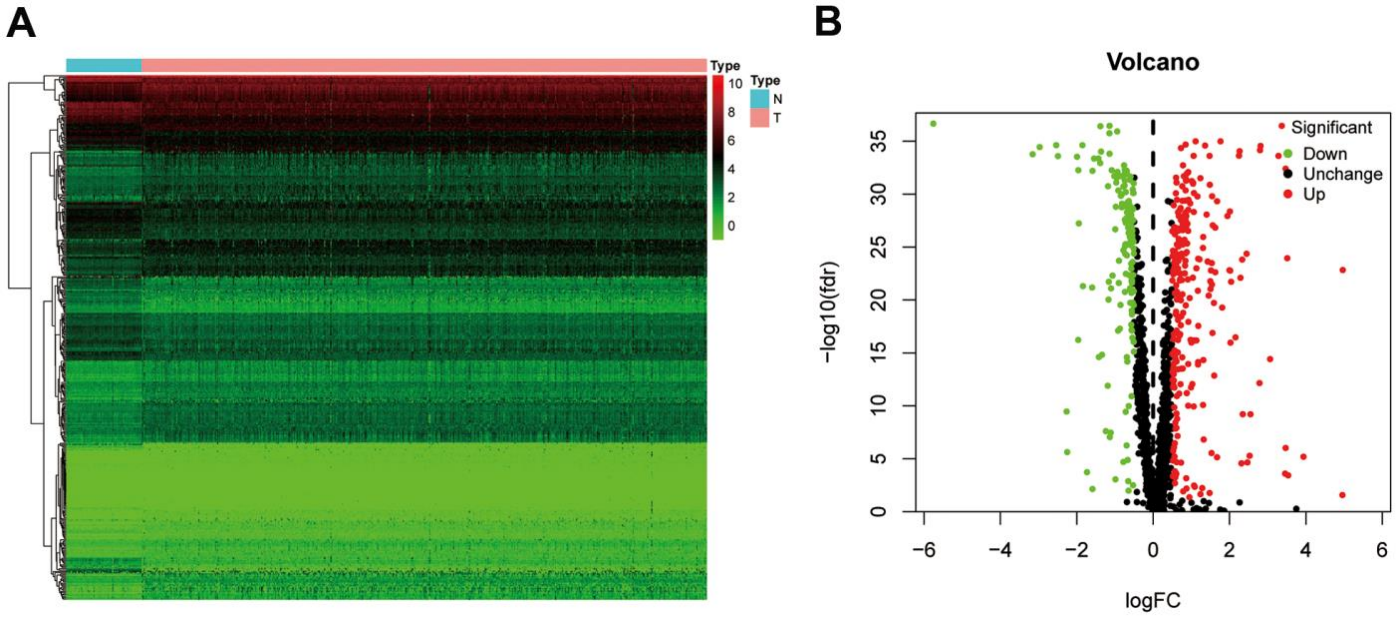
**The differentially expressed RBPs in ccRCC.** (**A**) Heatmap of differentially RBPs in different samples. Red represents upregulation and green represents downregulation. (**B**) Volcanic plot showing dysregulated RBPs in ccRCC tissue samples. ccRCC, clear cell renal cell carcinoma; RBPs, RNA-binding proteins.

The workflow of our study is presented in Figure 1. A total of 1542 RBP-related genes were processed using the “limma” package, and 397 complied with our standards (false discovery rate (FDR) < 0.05, |log2FC| > 0.5), including 153 significantly downregulated genes and 244 significantly upregulated genes. The results are displayed as heat maps and volcano plots (Figure 2A, 2B).

### Functional enrichment analysis of the differentially expressed RBPs

**Figure 3:**
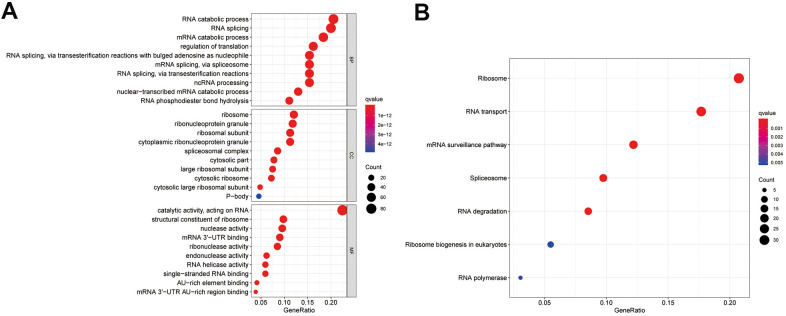
**Enrichment analysis of differentially expressed RBPs.** (**A**) Top 10 enriched BP terms, CC terms, MF terms. (**B**) The significant KEGG signal pathways. BP, biological process; CC, cellular components; MF, molecular functions; KEGG, Kyoto Encyclopedia of Genes and Genomes.

To investigate the mechanism of the differentially expressed RBPs, we conducted Gene Ontology (GO) and Kyoto Encyclopedia of Genes Genomes (KEGG) pathway enrichment analyses. According to the P value, from lowest to highest, the top three biological process (BP) terms were RNA catabolic process, mRNA catabolic process, and RNA splicing. In the molecular function (MF) category, the top three terms were catalytic activity acting on RNA, mRNA 3'-UTR binding, and ribonuclease activity. In the cellular component (CC) category, ribosomal subunit, ribonucleoprotein granule, and cytoplasmic ribonucleoprotein granule were the top-ranking terms. KEGG pathway analysis showed that these genes were closely related to the ribosome, RNA transport, and mRNA surveillance pathways. These results are presented in a bubble chart (Figure 3A, 3B).

### Protein-protein interaction (PPI) network construction and key module screening

**Figure 4:**
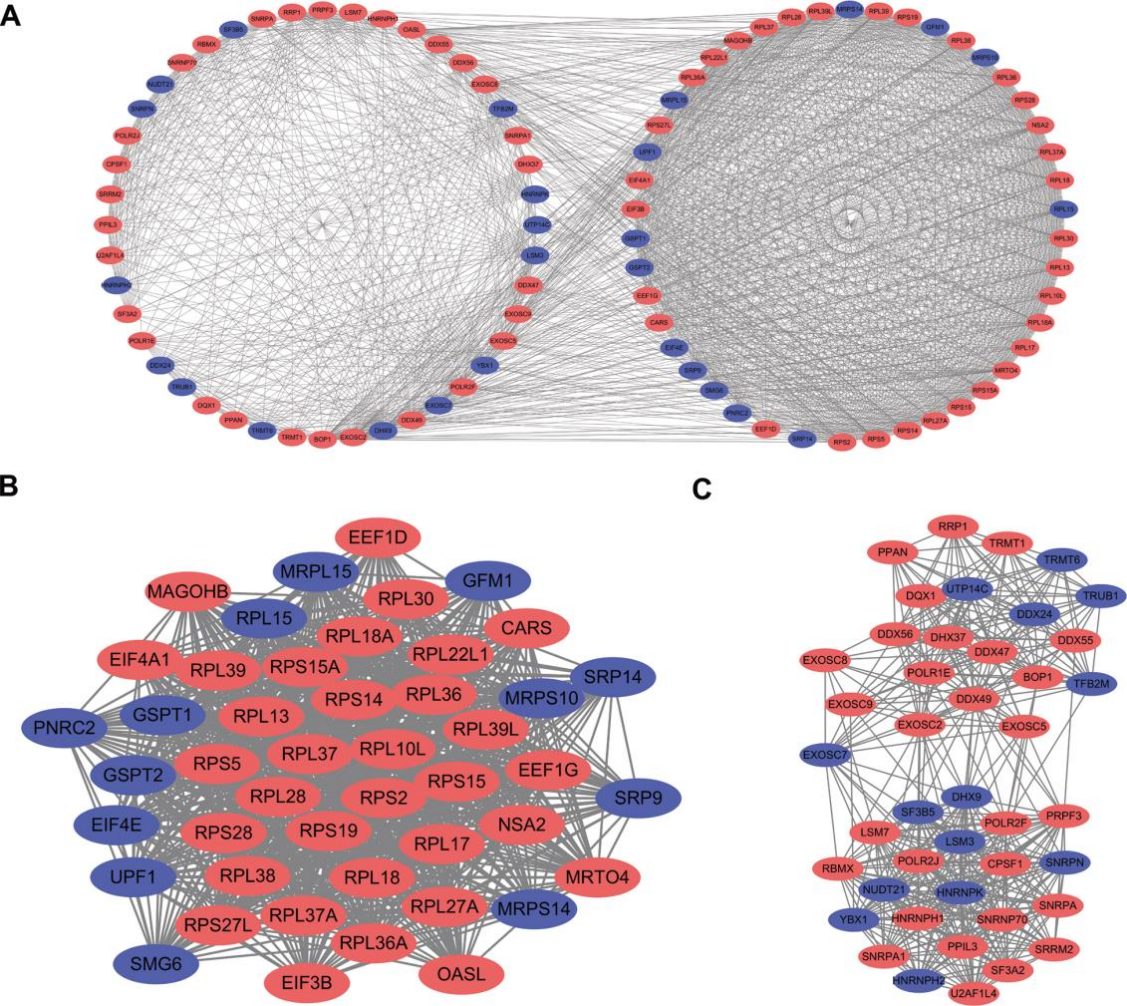
**PPI network construction and key module screening.** (**A**) Protein-protein interaction network. (**B**) Significant module 1. (**C**) Significant module 2. Blue: down-regulation genes. Red: up-regulation genes.

The PPI network was constructed to explore the underlying molecular biological function of these differentially expressed RBPs. A total of 398 nodes and 4067 edges were analyzed using plugin string. Then, we further analyzed the coexpression network to screen potential significant modules. Finally, the top two key modules were confirmed (Figure 4A). Module 1 consisted of 46 nodes and 905 edges (Figure 4B), and module 2 included 43 nodes and 429 edges (Figure 4C). Subsequently, the GO and KEGG pathway analysis revealed that the genes of module 1 were mostly enriched in the nuclear-transcribed mRNA catabolic process, ribosomal subunit, structural constituent of ribosome, and ribosome terms, whereas the genes in module 2 were primarily enriched in the RNA splicing, spliceosomal complex, catalytic activity acting on RNA, and spliceosome terms.

### Selection and verification of prognosis-related genes

**Figure 5:**
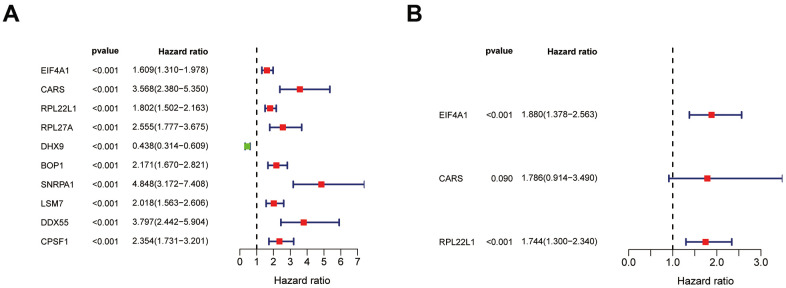
**Identification of prognosis related hub RBPs.** (**A**) Significance and Hazard ratio values of differentially expressed RBPs in univariate Cox regression. (**B**) Identification of prognosis related hub RBPs using multivariate Cox regression analysis.

**Figure 6:**
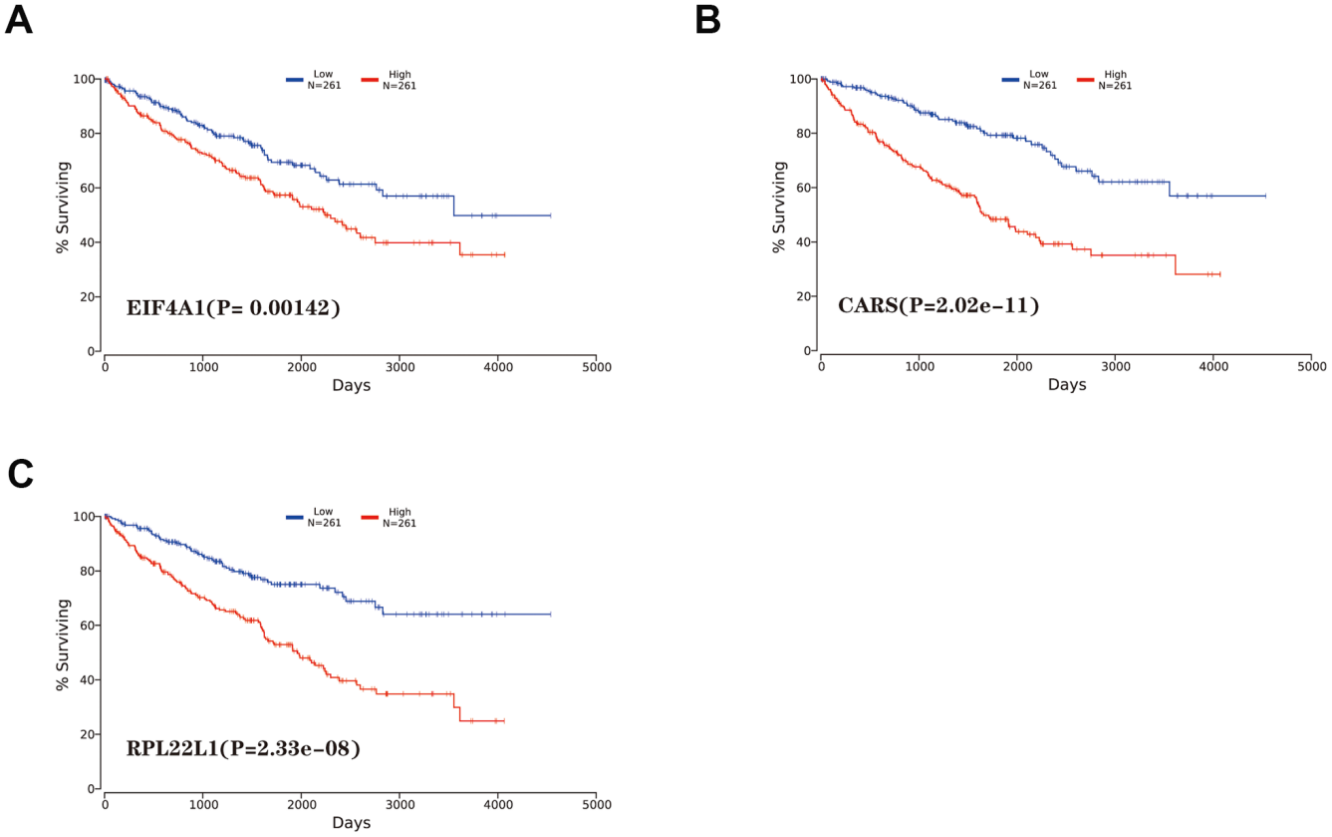
**Kaplan-Meier survival analysis of hub genes.** (**A**) EIF4A1. (**B**) CARS. (**C**) RPL22L1.

Among the 89 differentially expressed RBPs in the top two critical modules, 10 genes were identified as significantly related to prognosis by univariate Cox regression analysis (Figure 5A). Then, multivariate Cox regression was used to screen the RBP-related genes with independent prognostic value (Figure 5B), and three genes (EIF4A1, CARS, and RPL22L1) were selected as factors related to high risk in the prognostic model (Table 1). Additionally, we constructed a predictive model with these three genes in the training groups using LASSO regression analysis. Survival analyses were performed according to the expression of these three genes (Figure 6A–6C).

**Table 1:** List of the hub genes related with ccRCC patient prognosis.

**Gene name**	**Gene ID**	**Protein name**	**Location**	**Expression status**
EIF4A1	1973	Eukaryotic translation initiation factor 4A1	Chromosome 17	Up-regulated
CARS	833	Cysteinyl-tRNA synthetase	Chromosome 11	Up-regulated
RPL22L1	200916	Ribosomal protein L22 like 1	Chromosome 3	Up-regulated

**Figure 7:**
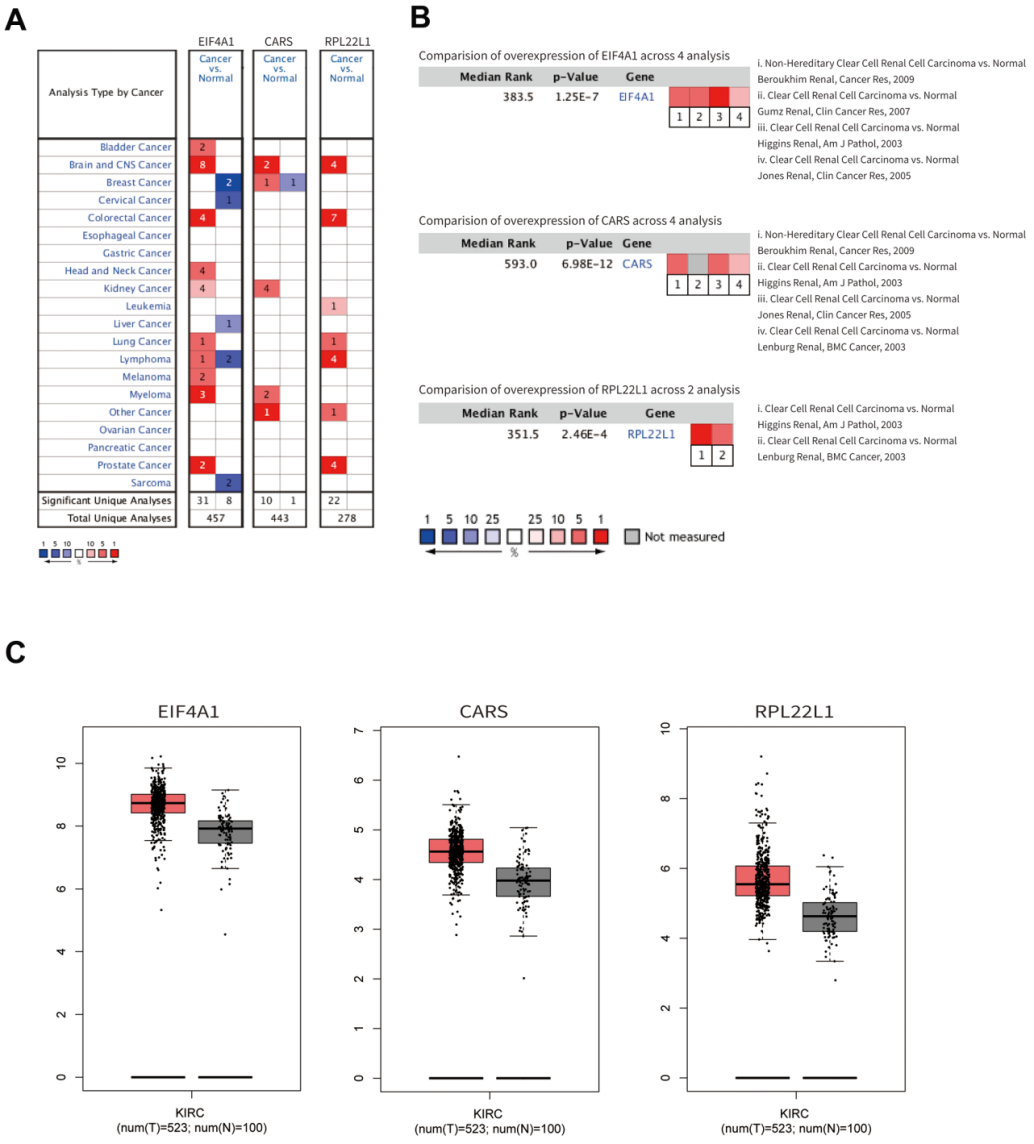
**The mRNA expression profiles of hub genes.** (**A**) The expression levels among different cancers. (**B**) The expression levels of the hub genes based on the published research. (**C**) The mRNA expression of hub genes in normal renal tissue and ccRCC.

**Figure 8:**
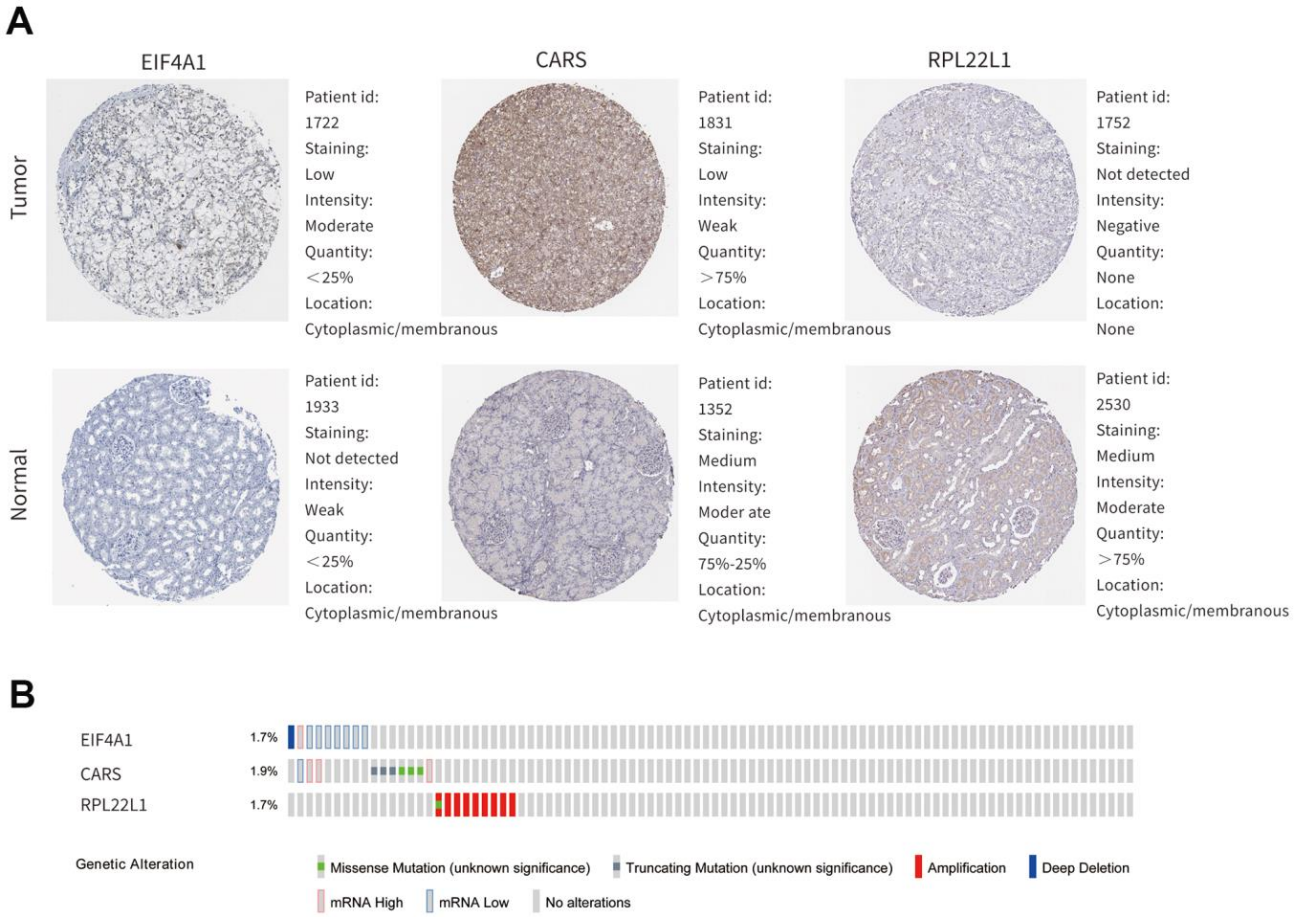
**The expression profiles of hub genes in online bioinformatics databases.** (**A**) The protein expression of hub genes in normal renal tissue and ccRCC on the HPA database. (**B**) Alterations of the hub genes on the cBioportal database.

As shown in Figure 7A–7C, the mRNA expression levels of EIF4A1, CARS, and RPL22L1 between renal tumor and normal tissues demonstrated that these genes were significantly upregulated in ccRCC patients (p < 0.001). Furthermore, these three genes were also significantly abnormally expressed at the protein level (Figure 8A). The higher magnified pictures of IHC staining were provided (Supplementary Figure 3A). In the Gene Alteration Atlas database, CARS possessed the most frequent genetic alteration, at a frequency of 1.9% among the samples; the other two genes also showed mutations (Figure 8B).

### Construction of the prognosis-related genetic risk score model

**Figure 9:**
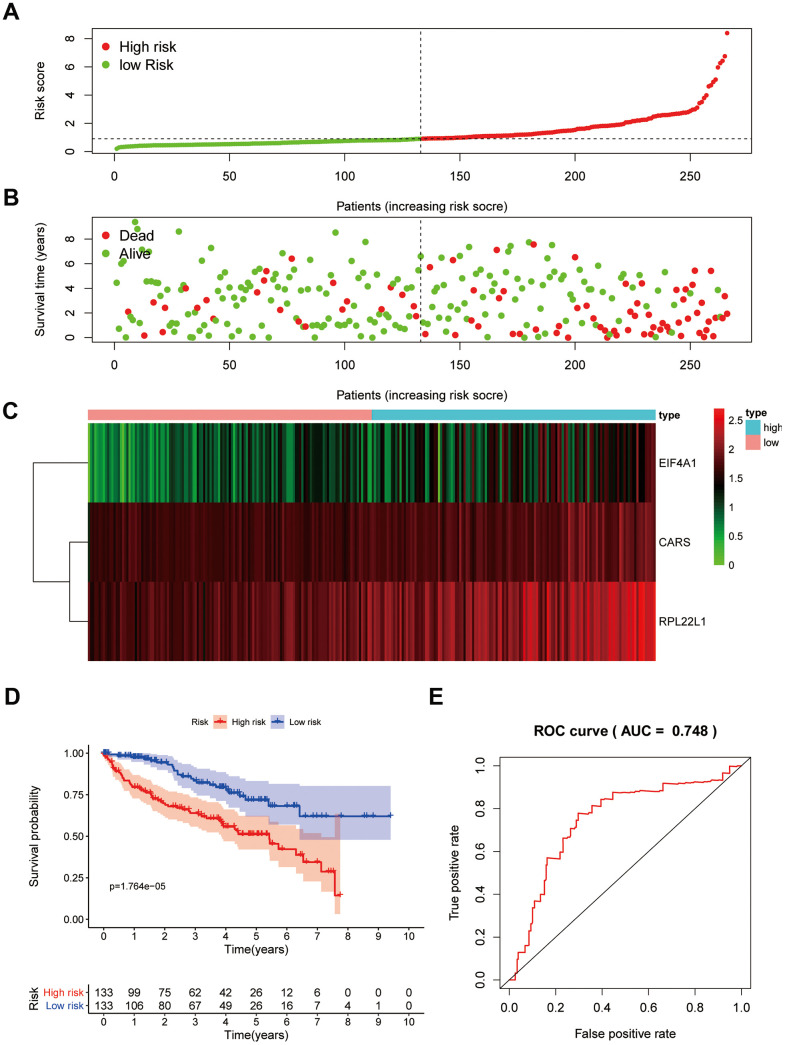
**Prognostic analysis of three-gene model in the training group.** The samples were divided into high- and low-risk subgroup according to the median of risk score. (**A**) The curve of risk score. (**B**) Survival status of patients. (**C**) Expression heatmap of three prognostic genes. (**D**) Survival curve for high- and low-risk subgroup. (**E**) ROC analysis of three-gene model. ROC, receiver operating characteristic.

Subsequently, we calculated the risk scores using the following formula based on their Cox coefficients for the training dataset: Risk score = 0.631*Exp_EIF4A1_ + 0.5801*Exp_CARS_ + 0.556*Exp_RPL22L1_. Then, ccRCC patients in the training group were split into a high-risk subgroup and a low-risk subgroup according to the median value of the risk score (Figure 9A). The dot plots show the survival status of the training group patients (Figure 9B). The expression heat maps of the three genes are shown in Figure 9C. Patients in the high-risk subgroup had considerably poorer overall survival (OS) than those in the low-risk subgroup (Figure 9D). Subsequently, receiver operating characteristic (ROC) analysis was performed to assess the predictive power of the three-RBP gene risk model; the area under the ROC curve (AUC) of this risk model for 5-year OS was 0.748 (Figure 9E). These results indicated that our risk model exhibited good specificity and sensitivity.

Then, ccRCC patients were stratified according to grade and stage. For all different stratifications, the OS time of the high-risk group was shorter than that of the low-risk group (Supplementary Figure 1). These results suggest that the RBP-related risk model for OS can predict the prognosis of ccRCC patients without the need to consider clinicopathological variables. Additionally, high expression of CARS and RPL22L1 was significantly correlated with late stage and high grade (p<0.001), whereas EIF4A1 expression was not correlated with stage and grade (Supplementary Figure 2).

### Verification of the risk score model in the testing group

**Figure 10:**
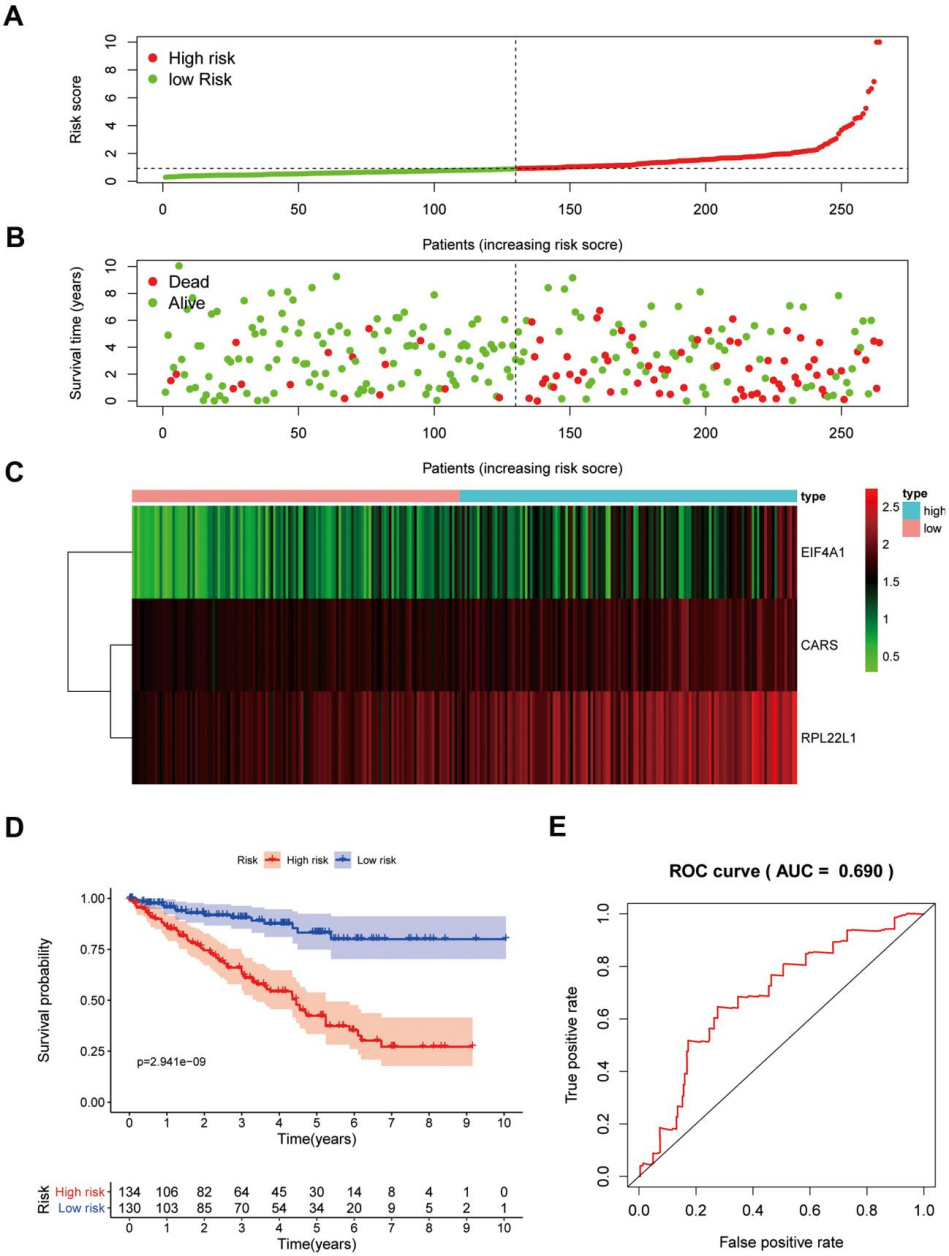
**Prognostic analysis of three-gene model in the testing group.** The samples were divided into high- and low-risk subgroup according to the median of risk score. (**A**) The curve of risk score. (**B**) Survival status of patients. (**C**) Expression heatmap of three prognostic genes. (**D**) Survival curve for high- and low-risk subgroup. (**E**) ROC analysis of three-gene model.

**Figure 11:**
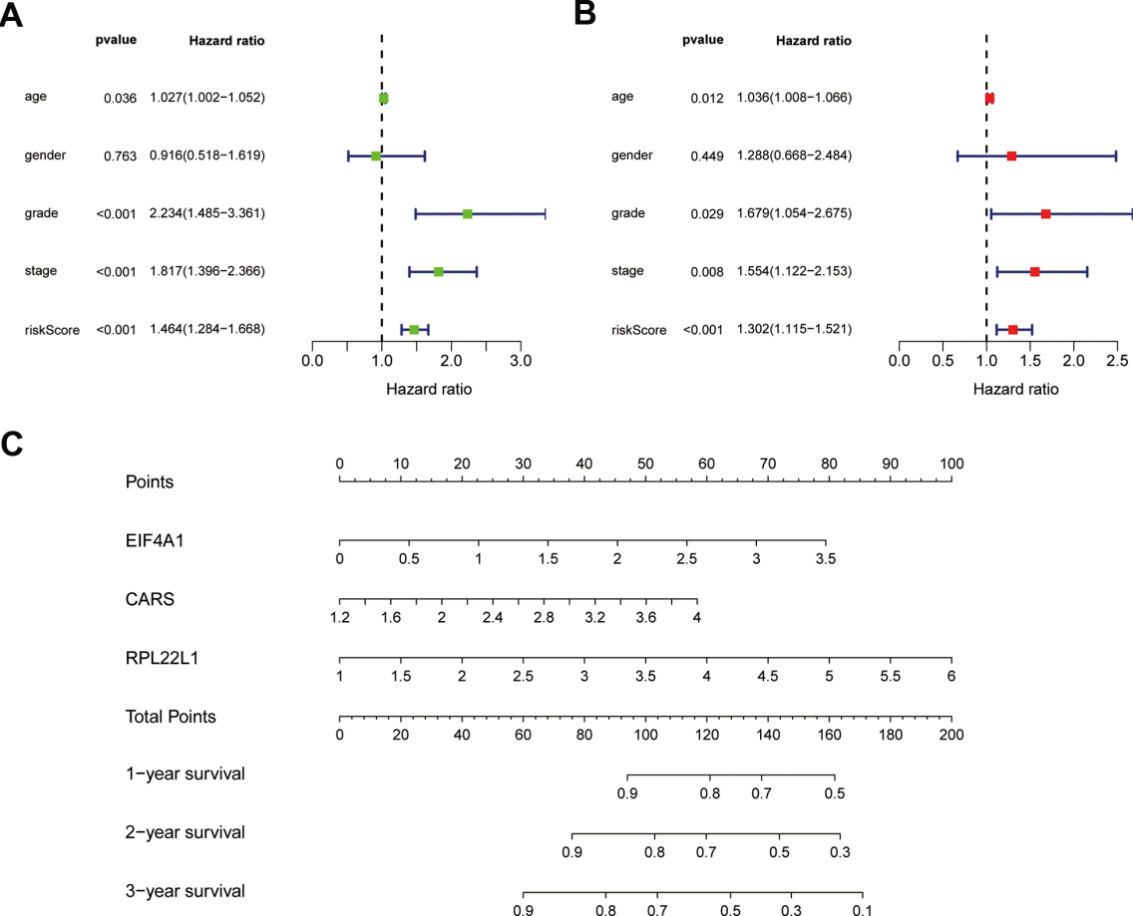
**Results of Cox regression for risk factors for ccRCC and construction of nomograms.** (**A**) Result of univariate Cox regression. (**B**) Result of multivariate Cox regression. (**C**) The nomograms for overall survival.

**Figure 12:**
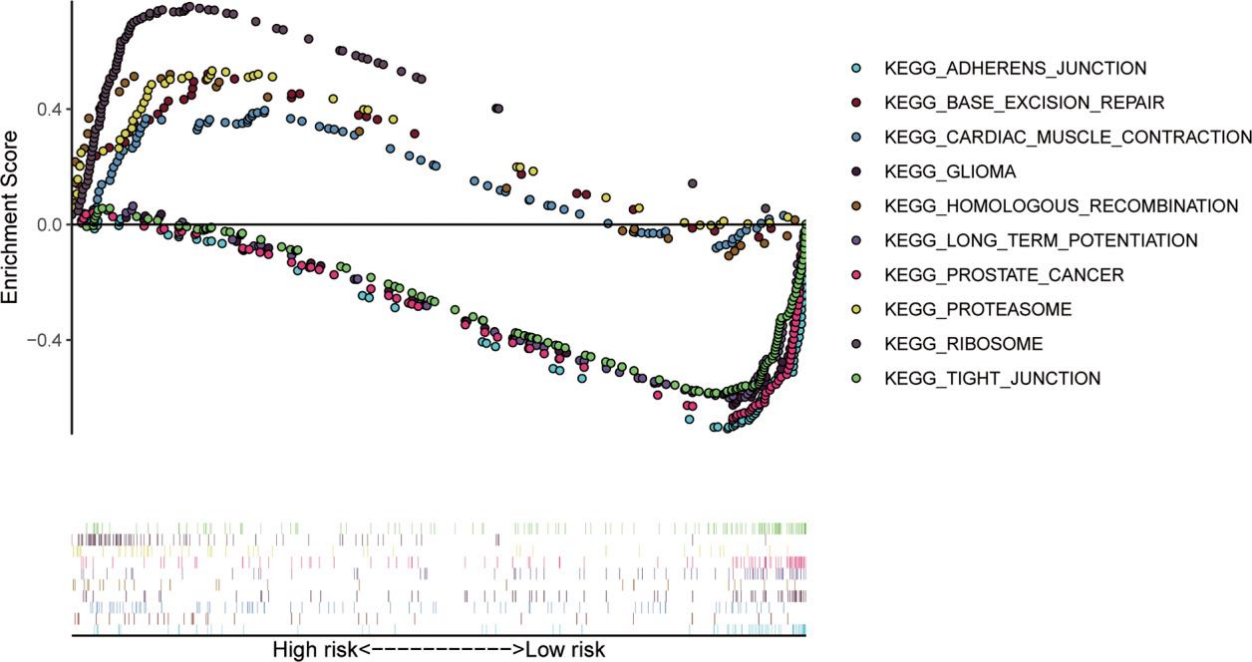
**The top 10 significant enriched KEGG pathways in the training group.**

To confirm that our risk model has similar predictive value in other ccRCC patient datasets, we performed the same analysis in the testing group. Patients in the testing group were divided into a high-risk subgroup and a low-risk subgroup according to the median risk score with the same risk assessment formula as the training group. The results demonstrated that high-risk patients exhibited worse outcomes than low-risk patients in the testing group (Figure 10A–10E). Furthermore, we analyzed the association of the risk score model and clinical features, such as sex, age, tumor grade, and stage, with patient survival. As displayed in Figure 11A, age, tumor stage, tumor grade, and risk score were related to the OS of patients using univariate Cox regression analysis. Next, we conducted multivariate Cox regression analysis, which demonstrated that tumor grade, tumor stage, and the risk score were significantly related to OS (Figure 11B). These results indicate that the risk score prognostic model can be used as an independent predictor of OS in ccRCC patients. Finally, we constructed a nomogram plot by combining the three-RBP signature with clinical characteristics, and this quantitative method can be used to assess the outcomes of ccRCC patients (Figure 11C). Gene set enrichment analysis (GSEA) was conducted to unravel the underlying molecular function of the risk model (Figure 12).

## DISCUSSION

Despite great progress in tumor diagnosis and treatment, the OS and mortality rates of ccRCC patients remain unsatisfactory [1, 15]. Therefore, understanding the pathological mechanism of ccRCC is critical to improving its survival rate. Several studies have demonstrated that RBPs promote the progression of various malignant tumors [16–18]. However, there is little data regarding the potential function of RBPs in ccRCC tumorigenesis.

In our current study, we screened 397 differentially expressed RBPs between normal and ccRCC tissues based on RNA sequencing information from the TCGA. Then, we comprehensively investigated their potential biological functions and built a PPI network based on these RBPs. We also conducted univariate and multivariate Cox regression analysis, ROC analysis, KM survival analysis and copy number alteration analysis of the key RBPs to comprehensively investigate their underlying biological roles and clinical value. Moreover, a risk model based on three prognosis-related RBPs was constructed to assess the prognosis of ccRCC patients. These studies may promote the development of diagnostic and therapeutic approaches for ccRCC patients.

Functional enrichment analysis of the differentially expressed RBPs suggested that these were mainly enriched in the RNA catabolic process, RNA splicing, regulation of translation, ribonucleoprotein granule, ribosome, spliceosomal complex, and mRNA 3'-UTR binding terms, which indicated that RBPs play significant roles as posttranscriptional regulators. During the last ten years, multiple studies have reported that RNA processing and metabolism are associated with the development and progression of human diseases [19–21]. RBP NONO promotes breast cancer cell growth by increasing the stability of STAT3 mRNA [22]. hnRNPL impacts the pathological conditions of membranous nephropathy by stabilizing the MTNR1A transcript [23]. In addition, SRSF3 inhibits B7-H3 expression by regulating in its RNA splicing in colorectal cancer [24]. Furthermore, the KEGG enrichment analysis demonstrated that the abnormally expressed RBPs participate in the progression of ccRCC by regulating ribosomes, RNA transport, spliceosomes, ribosome biogenesis in eukaryotes, RNA degradation, and polymerases.

Subsequently, a PPI network was constructed based on these abnormally expressed RBPs. We obtained 89 key RBPs from two key modules. Many of these key RBPs have been reported to exert significant effects on tumorigenesis and the development of tumors. EIF4A1, a eukaryotic translation initiation factor, plays vital roles in protein translation initiation, participates in epithelial-to-mesenchymal transition (EMT) and is related to a poor prognosis in patients with gastric cancer [25]. Another study showed that EIF4A1 contributes to gastric cancer progression by binding miR-1284 with high specificity [26]. CARS, cysteinyl-tRNA synthetase, boosts antitumor immunity by specifically interacting with TLR2/6 of antigen-presenting cells [27]. RPL22L1 is a ribosomal protein that regulates pre-mRNA splicing to control morphogenesis [28]. Numerous previous studies have demonstrated that the expression of RPL22L1 is aberrant in human cancer, and this feature is associated with a poor prognosis in malignant tumors [29–31]. Another study systematically analyzed the influence of RBPs by bioinformatics analyses of colorectal cancer metastasis, which provided novel insights for diagnosis and targeted therapy [32]. In addition, multiple published studies have identified promising risk models for predicting tumor patient prognosis. For example, two studies explored the prognostic value of RBPs using bioinformatics analyses and built risk models based on RBP-related genes in lung adenocarcinoma that play essential roles in predicting patient prognosis and developing novel therapeutic targets [33, 34]. In head and neck squamous cell carcinoma (HNSCC), a two-RBP gene signature (consisting of EZH2 and NOVA1) was constructed to predict the clinical prognosis of HNSCC patients, and this model will assist clinicians in providing a clinical diagnosis, individualized therapy, and prognostic assessment [35].

In our study, the key RBPs were selected using survival analyses and univariate and multivariate Cox regression analyses. We ultimately identified three RBPs related to the prognosis of ccRCC patients, including EIF4A1, CARS, and RPL22L1. The expression of these three key RBPs was verified at the transcription and translation levels using the Gene Expression Profiling Interactive Analysis (GEPIA) and Human Protein Atlas (HPA) databases, and the results indicated that these genes are upregulated in ccRCC tissues compared to normal tissues. These findings indicate that EIF4A1, CARS, and RPL22L1 may exert carcinogenic effects. Subsequently, we constructed an effective prognostic risk model based on these genes using LASSO regression analysis. Next, the ROC curve analysis showed the superior diagnostic accuracy of the three-gene risk model over clinical features alone, with an AUC of 74.8% in the training group and 69% in the test group. However, the pathological mechanism by which these three RBPs promote ccRCC tumorigenesis is still unclear, and further molecular function studies are necessary. A nomogram was constructed to assist clinicians in predicting the outcomes of ccRCC patients. Our prognostic nomogram based on three RBPs may provide more reliable information for individualized treatment than conventional clinical characteristics.

In summary, this study provides new insights into the roles of RBPs during ccRCC development and reports the construction of an effective prognostic risk model to evaluate patient prognosis. In addition, RBP-related genes play a vital role in tumorigenesis, which suggests that these key RBPs may provide additional clues to aid in the exploration of new diagnostic strategies. However, there are some limitations to our study. First, the prognostic model was built based only on expression data from the TCGA. Although all samples were classified into a training group and testing group and the predictive efficacy of the prognostic model was verified in the testing group, our outcome would be more convincing with verification in other public datasets. We will collect the expression profiles and corresponding clinical data of patients from our own research center to complete this work. Second, our conclusions are based on a retrospective analysis, and these outcomes should be verified in prospective clinical research. Finally, the biological roles of the key RBPs in this risk model must be further investigated in ccRCC.

Overall, we systematically investigated the biological functions and prognostic value of differently expressed RBPs by comprehensive bioinformatics analysis in ccRCC. A prognostic signature consisting of three RBP-coding genes was established to serve as a novel prognostic factor for ccRCC. To the best of our knowledge, this study is the first to develop an RBP-related prognostic model for ccRCC. Our results contribute to the identification of novel diagnostic and prognostic molecular markers.

## MATERIALS AND METHODS

### Source of data

The RNA sequencing data of 539 ccRCC samples and 72 normal renal samples and the corresponding clinical features of the patients were extracted from the TCGA (https://tcga-data.nci.nih.gov/tcga/). Patients who lacked information on age, sex, and survival status were excluded from the subsequent analysis. Finally, 530 ccRCC tumor samples and 72 normal samples were screened from the TCGA database. R language (version 3.6.0) was used to process the data.

### Identification of differentially expressed RBPs and functional enrichment analysis

The Wilcox signed-rank test was used to screen the differentially expressed RBPs, with FDR less than 0.05 and absolute fold change (FC) more than 0.5 as the cutoffs. The functional enrichment analysis included KEGG and GO analyses, which were applied to evaluate the functional categories related to the differentially expressed RBPs using the R package “clusterProfiler”; FDR less than 0.05 was set as the significance threshold. The results were visualized with the R package ggplot2. Then, a PPI network of the differentially expressed RBPs was built using the Search Tool for the Retrieval of Interacting Genes/Proteins (STRING) database (https://string-db.org/). We imported these data into Cytoscape (version 3.6.1). The key modules were screened by using the MCODE plugin in Cytoscape.

### External validation of the prognostic signature

The mRNA expression levels of the key genes between renal tumors and normal samples were obtained from the GEPIA online database (http://gepia.cancer-pku.cn/). Differential protein expression between renal tumor and normal tissues was assessed with data from the HPA database (https://www.proteinatlas.org/). Then, mutation data and overall copy number alteration data were obtained for hub genes in the cBioPortal database (http://www.cbioportal.org/). We validated the significance and the expression rank of hub RBPs in the Oncomine database (https://www.oncomine.org/). Kaplan-Meier survival analysis was performed with the OncoLnc online software (http://www.oncolnc.org/). GSEA was conducted with online software (version 4.0.3).

### Construction and validation of the RBP-related prognostic model

A total of 530 ccRCC tumor samples were randomly classified into the training group (n=266) and the testing group (n=264). The detailed clinical data of ccRCC patients are shown in Table 2. Prognosis-related RBP genes associated with patient OS were screened using univariate Cox regression. With the cutoff values of Cox P < 0.00001 and KM P < 0.00001, ten differentially expressed genes (DEGs) were considered survival-related RBP genes. Subsequently, LASSO Cox regression was used to build the RBP-related prognostic risk model from the training group data. The LASSO Cox regression model was performed using the “glmnet” R package. The prognostic risk signature includes a Cox regression coefficient and can be used to calculate the risk scores for all samples. The formula of the prognostic risk model was given as risk score = expression level of gene 1*α1 + expression level of gene 2*α2 +... +expression level of gene n*αn, in which α is representative of the Cox regression coefficient of each parameter.

**Table 2:** Clinical parameters of 530 ccRCC patients.

**Clinical parameters**	**Total**	**Training group**	**Testing gruop**
	**(n=530)**	**(n=266)**	**(n=264)**
Age(year, mean±SD)	60.56±12.14	60.52±12.24	60.61±12.06
Gender(n,%)			
Male	344(64.9)	175(65.7)	169(64.0)
Female	186(35.1)	91(34.3)	95(36.0)
Tumor grade(n,%)			
G1	14(2.6)	5(1.9)	9(3.4)
G2	227(42.8)	106(39.8)	121(45.8)
G3	206(38.9)	114(42.9)	92(34.8)
G4	75(14.2)	36(13.5)	39(14.8)
Gx	5(1.0)	3(1.1)	2(0.8)
Unknown	3(0.5)	2(0.8)	1(0.4)
Pathological stage(n,%)			
Stage I	265(50.0)	132(49.6)	133(50.4)
Stage II	57(10.8)	33(12.4)	24(9.1)
Stage III	123(23.2)	62(23.3)	61(23.1)
Stage IV	82(15.5)	37(13.9)	45(17.0)
Unknown	3(0.5)	2(0.8)	1(0.4)
AJCC T(n,%)			
T1	271(51.1)	136(51.1)	135(51.1)
T2	69(13.0)	36(13.6)	33(12.5)
T3	179(33.8)	87(32.7)	92(34.9)
T4	11(2.1)	7(2.6)	4(1.5)
AJCC N(n,%)			
N0	239(45.1)	123(46.2)	116(43.9)
N1	16(3.0)	9(3.4)	7(2.7)
Nx	275(51.9)	134(50.4)	141(53.4)
AJCC M(n,%)			
M0	420(79.2)	215(80.8)	205(77.7)
M1	78(14.7)	36(13.5)	42(15.9)
Mx	30(5.7)	13(4.9)	17(6.4)
Unknown	2(0.4)	2(0.8)	-
Survival status(n,%)			
Alive	364(68.7)	182(68.4)	182(68.9)
Dead	166(31.3)	84(31.6)	82(31.1)
Survival months(mean±SD)	37.88±27.20	36.59±26.73	39.19±27.64
Risk scores(mean±SD)	1.31±1.32	1.29±1.15	1.33±1.46
High(n,%)	267(50.4)	133(50.0)	134(50.8)
Low(n,%)	263(49.6)	133(50.0)	130(49.2)

ccRCC: clear cell renal cell carcinoma; SD: Standard Deviation; AJCC: American Joint Committee on Cancer.

After calculating the risk scores, the ccRCC patients were assigned to high- and low-risk subgroups. We compared the difference in survival rates among the two risk subgroups by the log-rank test in the R environment. Additionally, a ROC curve was plotted to assess the prognostic accuracy of the predictive model using the “survivalROC” R package. Further, the prognostic value of this model was evaluated using univariate and multivariate Cox regression analyses. These analyses were performed with the “survival” and “survminer” R packages. The testing group was used as a validation set to evaluate the predictive accuracy of this model by the same methods. Finally, we established a nomogram to visualize model efficiency using the rms R package.

### Statistical analysis

The data were analyzed using the PERL programming language (version 5.32.0). All statistical analyses were conducted using the R language (version 3.6.0). Differences with P < 0.05 were regarded as statistically significant.

## Supplementary Material

Supplementary Figures
