# Supplementary material for: Development of prognostic signature based on RNA binding proteins related genes analysis in clear cell renal cell carcinoma
Source: Aging (Albany NY). 2021 Jan 10;13(3):3926–44. doi: 10.18632/aging.202360 (PMC7906138; doi:10.18632/aging.202360)
Supplement: Supplementary Figures [file aging-13-202360-s001.pdf]

SUPPLEMENTARY FIGURES

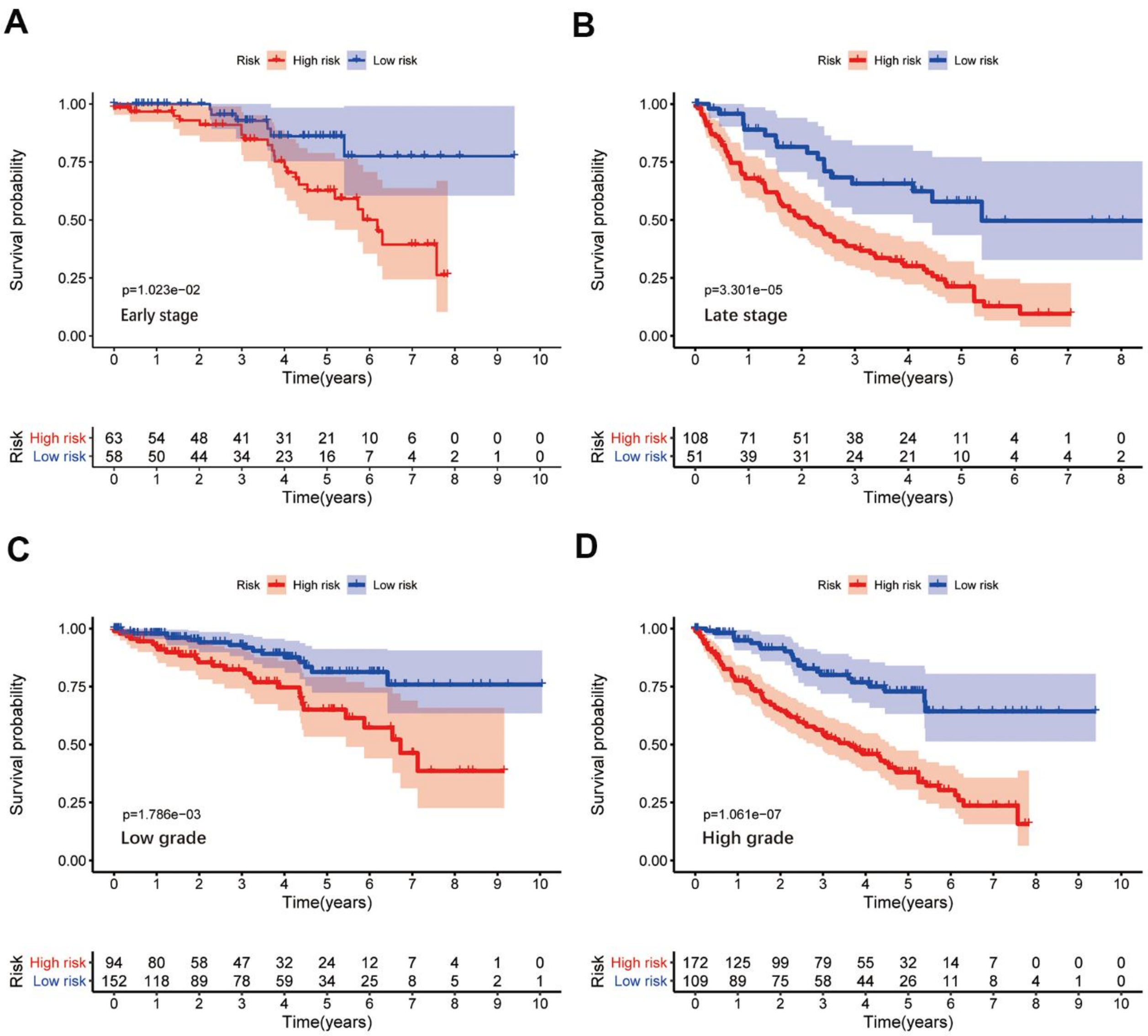

Supplementary Figure 1. Kaplan-Meier survival curves for the high- and low-risk groups stratified by clinicopathological variables. (**A**, **B**) Stage. (**C**, **D**) Grade.

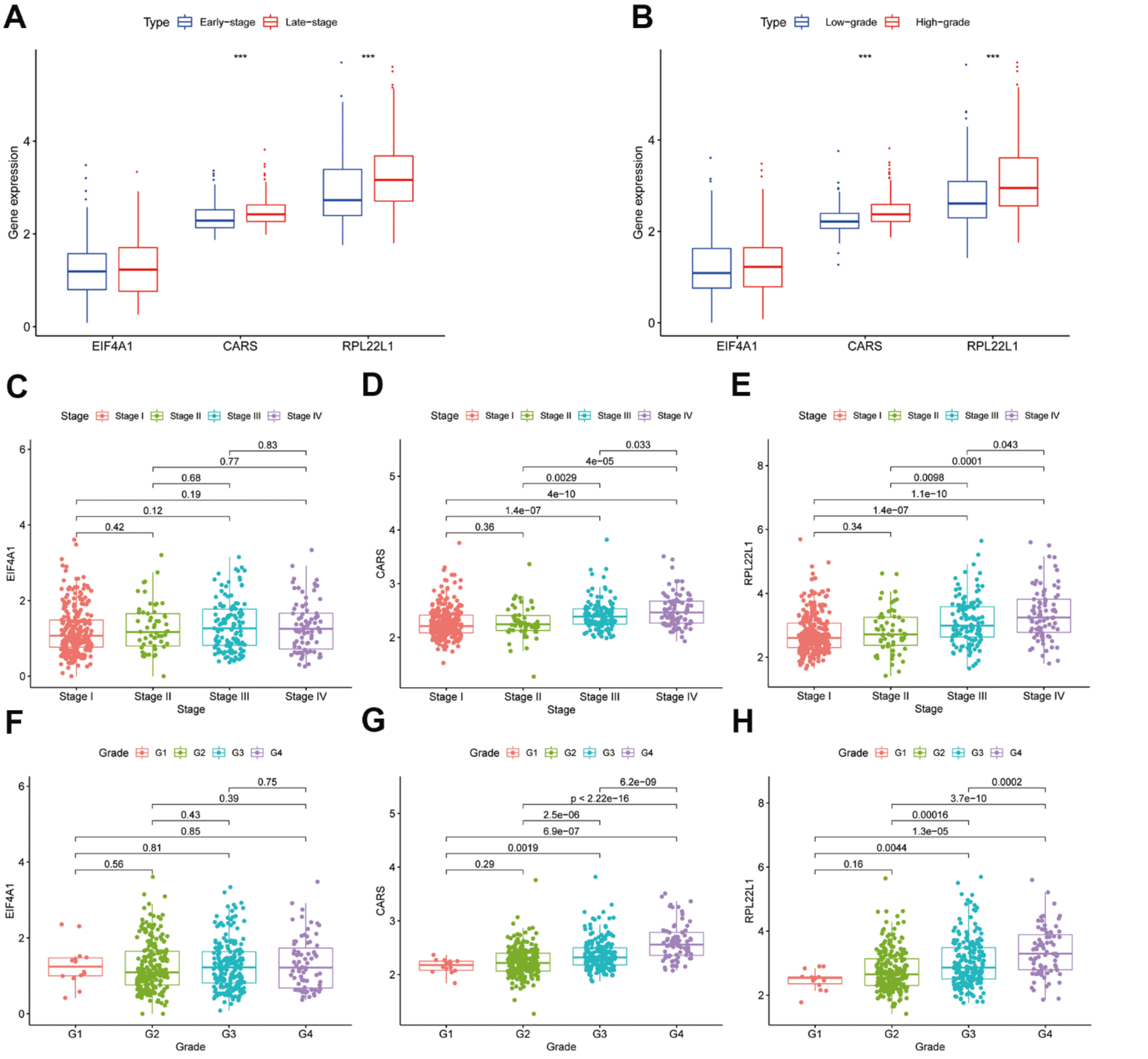

Supplementary Figure 2. The mRNA expression profiles of three hub RBPs stratified by clinicopathological variables. (**A**, **C**–**E**) Stage. (**B**, **F**–**H**) Grade

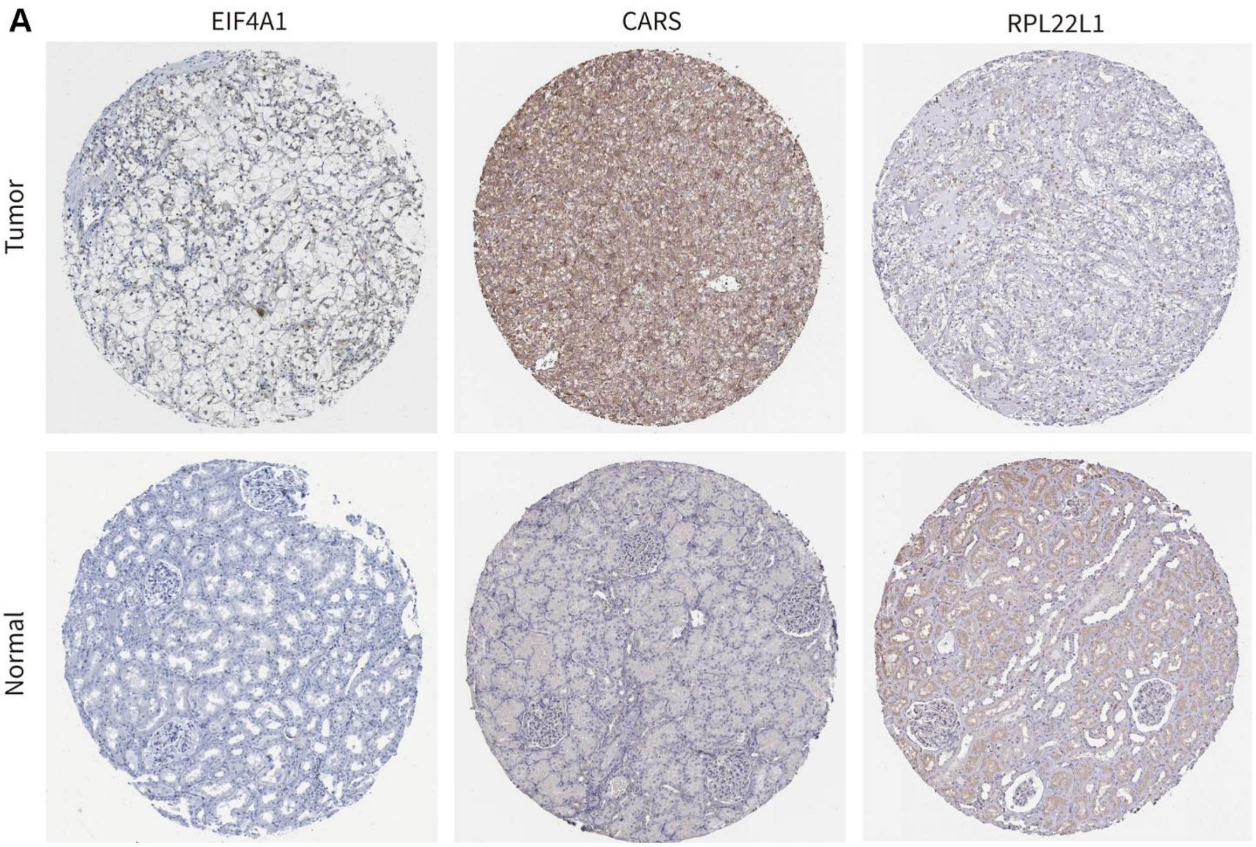

Supplementary Figure 3. The protein expression of hub genes in normal renal tissue and ccRCC on the HPA database. (**A**) The IHC staining of EIF4A1, CARS, and RPL22L1.
